# Optimal generation of spatially coherent soft X-ray isolated attosecond pulses in a gas-filled waveguide using two-color synthesized laser pulses

**DOI:** 10.1038/srep38165

**Published:** 2016-12-08

**Authors:** Cheng Jin, Kyung-Han Hong, C. D. Lin

**Affiliations:** 1 Department of Applied Physics, Nanjing University of Science and Technology, Nanjing, Jiangsu 210094, P. R. China; 2 Department of Electrical Engineering and Computer Science and Research Laboratory of Electronics, Massachusetts Institute of Technology (MIT), Cambridge, Massachusetts 02139, USA; 3 J. R. Macdonald Laboratory, Department of Physics, Kansas State University, Manhattan, Kansas 66506, USA

## Abstract

We numerically demonstrate the generation of intense, low-divergence soft X-ray isolated attosecond pulses in a gas-filled hollow waveguide using synthesized few-cycle two-color laser waveforms. The waveform is a superposition of a fundamental and its second harmonic optimized such that highest harmonic yields are emitted from each atom. We then optimize the gas pressure and the length and radius of the waveguide such that bright coherent high-order harmonics with angular divergence smaller than 1 mrad are generated, for photon energy from the extreme ultraviolet to soft X-rays. By selecting a proper spectral range enhanced isolated attosecond pulses are generated. We study how dynamic phase matching caused by the interplay among waveguide mode, neutral atomic dispersion, and plasma effect is achieved at the optimal macroscopic conditions, by performing time-frequency analysis and by analyzing the evolution of the driving laser’s electric field during the propagation. Our results, when combined with the on-going push of high-repetition-rate lasers (sub- to few MHz’s) may eventually lead to the generation of high-flux, low-divergence soft X-ray tabletop isolated attosecond pulses for applications.

Powerful coherent soft X-ray isolated attosecond pulses (IAPs) are highly in demand for attosecond experiments^1^, for example, in attosecond-pump/attosecond-probe measurements of electronic processes in atoms and molecules, for time-resolved structural dynamics of biomolecules, and for high-contrast biological imaging. At present, IAPs are mainly produced by high-order harmonic generation (HHG) in gases. The generation of IAP is an extreme nonlinear process occurring only during a fraction of the driving laser period, in which tunnel-ionized electrons recollide and recombine with atomic ions to emit high-energy photons. For the generation of IAPs, few-cycle laser pulses are preferably used since multi-cycle laser pulses would generate attosecond pulse trains (APTs).

Under the scenarios that high harmonics are generated from only half an optical cycle of an infrared laser pulse, several techniques have been developed for the production of IAPs in the extreme ultraviolet (XUV). For instance, using amplitude gating^2^ IAP as short as 80 attoseconds was reported with carrier-envelope-phase (CEP) stabilized few-cycle pulses. Similarly, polarization gating was used to produce an isolated 130-as pulse^3^,^4^ and double optical gating (DOG)^5^ was used to generate a 67-as IAP. Based on phase matching achieved for different ranges of harmonics, spatiotemporal gating was applied in a tight-focusing geometry to generate IAP^6^,^7^. Other approaches include ionization gating^8^,^9^, optimizing pressure and length of a gas cell^10^,^11^, using a spatial filter in the far field^12^,^13^,^14^,^15^, or two-color synthesis to produce gigawatt-scale IAPs^16^,^17^. Recently the technique of attosecond lighthouse or photonic streaking employing wavefront rotation scheme has also been demonstrated^18^,^19^. Beyond the XUV regime, Chen *et al*. showed the generation of isolated soft X-ray attosecond pulses at photon energies up to 180 eV by using a 2-*μ*m, 10-cycle driving laser^20^. Based on spatiotemporal isolation of wavefront rotation, Biegert’s group reported continuous photons up to the carbon K-edge of 284 eV with pulse duration below 400 as and with a bandwidth supporting a 30-as pulse duration^21^. The same group also reported a 0.5-keV soft X-ray supercontinuum which may support a 13-as IAP if the pulse is transform-limited, using a CEP-stabilized, 1.8-*μ*m few-cycle laser pulse^22^. The polarization gating technique has been recently implemented to generate super-continuous HHG spectra in the water window with a two-cycle, 1.7-*μ*m laser by Chang’s group^23^. In addition, Stein *et al*. reported the generation of soft X-ray harmonics extending to 450 eV in a neon-filled semi-infinite gas cell by a kHz, 2.1-*μ*m mid-infrared optical parametric chirped-pulse amplification (OPCPA) source^24^. In the soft X-ray region, however, characterization of attosecond pulses has not been established yet since the central momentum approximation used in FROG-CRAB phase retrieval is not applicable to these broadband IAP’s.

While soft X-ray harmonics have been generated with mid-infrared lasers, they have large angular divergence^25^,^26^ and their intensities are limited by low conversion efficiency. How to generate useful intense soft X-ray IAPs in the laboratory is an important question. In principle one can improve the efficiency either by enhancing harmonics generated from individual atoms by modifying the sub-cycle waveform^16^,^17^,^27^,^28^,^29^,^30^,^31^,^32^,^33^,^34^,^35^,^36^,^37^,^38^, or by optimizing macroscopic phase-matching conditions^39^,^40^,^41^. We have recently proposed different schemes to enhance single-atom harmonic yield by optimally synthesizing multi-color sinusoidal laser pulses^42^,^43^,^44^. Optimization of the waveform was derived in such a way that harmonics generated are better phase-matched in the gas medium. We have also demonstrated such optimized two-color waveform in a hollow waveguide can efficiently generate low-divergence soft X-ray harmonics when the gas pressure and waveguide parameters^45^ were optimized.

In this work we apply the same technique to study the generation of soft X-ray IAPs. For this purpose we choose the previously optimized two-color waveform that consists of the fundamental laser and its second harmonic^42^ in a gas-filled hollow waveguide. Intense second harmonic is easily obtained in the laboratories. It can be combined with the fundamental to alter the half-cycle periodicity. In a hollow waveguide, constant laser intensity can be maintained over an extended distance. The geometric phase of the laser beam does not vary with the radial distance. Harmonic generation experiments in a waveguide have been widely used^46^,^47^,^48^ to create quasi-phase matching (QPM) conditions^49^,^50^, to generate soft X-ray high harmonics^51^,^52^ or keV-harmonics with mid-infrared lasers^40^,^53^,^54^.

Our goal in this work is to demonstrate how spatially coherent soft X-ray IAPs are generated at the optimal macroscopic conditions by an optimized two-color waveform^42^ in a hollow waveguide. We will show that high harmonics from XUV to soft X-rays have low divergence (smaller than 1 mrad) at the optimized gas pressure and waveguide parameters, where their yields are greatly enhanced in comparison with the single-color laser. We will demonstrate the phase matching mechanism for the generated IAP by analyzing the two-color waveform during its propagation in the medium. The paper is arranged as follows: In “Methods”, we will briefly summarize the propagation equations in a hollow waveguide, the wavelet theory for time-frequency analysis, and formulas for the generation of macroscopic attosecond pulses. In “Results”, simulated high harmonic spectra and IAPs synthesized in different spectral ranges will be presented. Both the evolution of the electric field and the time-frequency harmonic emission along the propagation direction will be presented to help understanding the macroscopic IAP generation. A short summary in “Discussion” will conclude this paper.

## Methods

### Propagation equations of the driving laser pulse and harmonic field in a gas-filled waveguide

To calculate harmonic radiation generated in a macroscopic medium, time-dependent Schrödinger equations (TDSEs) and coupled Maxwell’s wave equations (MWEs)^55^,^56^,^57^,^58^,^59^,^60^ are solved to account for single-atom response to the driving pulse and the propagation of radiation in the gas medium. The solution of TDSE for the single atom response is replaced by the quantitative rescattering (QRS) model^61^,^62^,^63^. Details of propagation equations of the driving pulse and high-harmonic fields have been presented in ref. 64. Here we only discuss how to solve the key equations in a gas-filled hollow waveguide.

In a reference frame moving at the speed of light *c* (*z*′ = *z* and *t*′ = *t* − *z*/*c*), the MWE for the driving laser in the frequency domain is


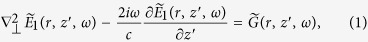


where


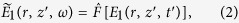


and


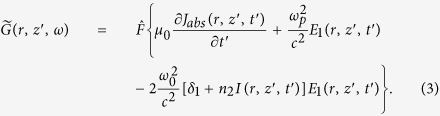


Here 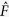 is the Fourier transform operator acting on the temporal coordinate. In Eq. (3), absorption (*J*_*abs*_) due to ionization, atomic dispersion (*δ*_1_), Kerr nonlinearity (*n*_2_), and plasma effect are all included. The plasma frequency is 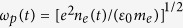, where *m*_*e*_ and *e* are the electron mass and charge, respectively, and *n*_*e*_(*t*) is the density of free electrons.

The MWE for the emitted high-harmonic field can be written as


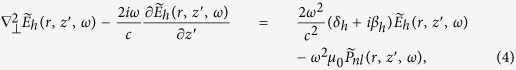


where


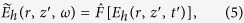


and


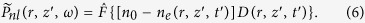


Here *n*_0_ is the neutral atom density, and *D*(*r*, *z*′, *t*′) is the single-atom induced dipole moment. *δ*_*h*_ and *β*_*h*_ account for the dispersion and absorption of the medium on the harmonics, respectively.

To solve Eqs (1) and (4) the operator-splitting method is used. The advance of electric field from *z*′ to 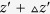 is separated into two steps as shown in the following^65^:


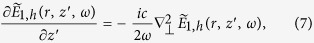



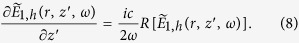


Here 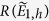 stands for all linear and nonlinear terms on the right-hand sides of Eqs (1) and (4).

In order to impose the boundary conditions of the hollow waveguide, 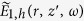 is written as a superposition of eigenmodes^66^,^67^,^68^:


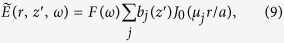


where *μ*_*j*_ are the roots of Bessel function of the first kind *J*_0_(*μ*_*j*_) = 0, and *a* is the hollow-core radius. Inserting Eq. (9) into Eq. (7), and using the orthonormal relation 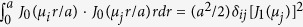, for each *ω*, we obtain


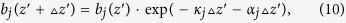


where the propagation constant *κ*_*j*_ is^69^


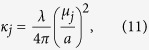


and the mode loss term is^69^


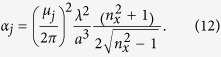


Here *n*_*x*_ is the refractive index of the cladding, and *λ* = 2*πc*/*ω*. Note that the minus sign before 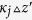 in Eq. (10) is different from that in refs 67 and 68 because of the different convention for the Fourier transform.

In the calculations, we assume the spatial distributions of the two colors at the entrance of the hollow waveguide are the lowest EH_11_ mode. This can be achieved by adjusting the ratio between the beam waist of the incident laser and the radius of the waveguide to be about 65%. High harmonics emitted on the exit plane of the hollow waveguide are taken as the near-fields.

### Far-field harmonic emission

Near-field high harmonics may propagate further in the vacuum till they are detected. The far-field harmonic can be obtained from the near-field through a Hankel transformation^64^


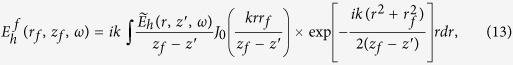


where *J*_0_ is the zero-order Bessel function, *z*_*f*_ is the far-field position from the laser focus, *r*_*f*_ is the transverse coordinate in the far field, and the wave vector *k* is given by *k* = *ω*/*c*. Note that the optical path difference at different radial distance *r*_*f*_ can be eliminated by using the convex lens experimentally. Mathematically, this can be carried out by multiplying a phase factor 
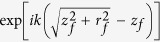
 in Eq. (13).

### Wavelet analysis of attosecond pulses

Time-frequency analysis of harmonic emission is carried out by using the wavelet transform^70^,^71^,^72^,^73^,^74^:


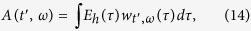


with the wavelet kernel 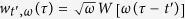. We use the Morlet wavelet^70^:


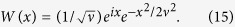


*ν* is chosen to be 15 in this paper.

To avoid the complexity of analyzing the full spatial distribution of the harmonics in the near field, we calculate *A*(*t*′, *ω*) at each radial point and then integrate over the radial coordinate^15^,^72^:


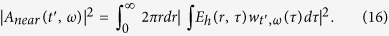


A spectral filter is then used to select a range of harmonics (*ω*_1_ – *ω*_2_) to generate attosecond pulses. The total intensity of attosecond pulses in the near field is obtained from^15^,^75^:


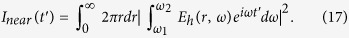


In the far field, a spatial filter is employed to select harmonics with a prescribed area. We assume that the filter is circular with a radius *r*_0_, and is perpendicular to the propagation direction. The intensity of attosecond pulses in the far field is^15^


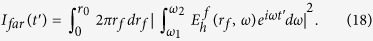


## Results

In the calculations, the two colors are both assumed to have Gaussian temporal envelope with the full width at half maximum (FWHM) duration of 16 fs (3 cycles of the 1.6-*μ*m laser). The target is neon gas and the desnity distribution is uniform within the hollow waveguide. The initial on-axis electric field is the optimized two-color waveform obtained from Supplementary Table 5 in ref. 42, in which the 1.6- and 0.8-*μ*m lasers have peak intensity of 3.0 and 0.5 × 10^14^ W/cm^2^, respectively, and the 0.8-*μ*m laser has the relative phase of 0.34 *π*.

### High harmonics generated with the optimized two-color waveform in the near and far field

**Figure 1:**
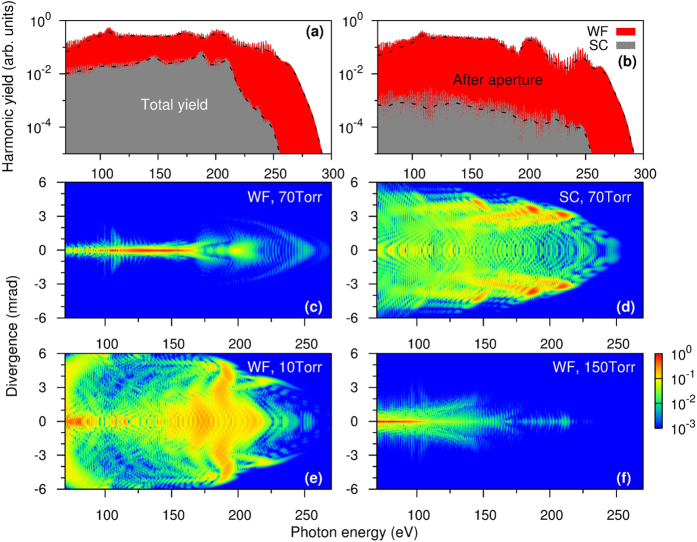
(**a**) Total harmonic yields at the exit face of the hollow waveguide and (**b**) harmonic yield integrated within 1 mrad using an aperture in the far field, by two-color (1.6 + 0.8 *μ*m) waveform (WF) and single-color (SC) laser. The gas pressure is 70 Torr. (**c**) and (**d**) Divergence of the far-field harmonics generated by two-color WF and by SC pulses, respectively. Divergence of harmonics generated by the two-color WF at gas pressure of 10 Torr (**e**) and 150 Torr (**f**), respectively. Length (radius) of the waveguide is 5 mm (125 *μ*m).

We first show the total harmonic yield at the exit of the waveguide (near field) generated by the optimized waveform (WF) in Fig. 1(a). In the simulation, the radius of the waveguide was fixed as 125 *μ*m (a two-color Gaussian beam with the pulse energy of 0.62 mJ is incident into the waveguide). We varied the length of the waveguide and the gas pressure to achieve highest yield at the cutoff energy of about 250 eV (close to the cutoff of single-atom response). In other words, the optimization goal here is to reach best balance between harmonic yield and cutoff energy. We found the optimal values of the waveguide length and the pressure were 5 mm and 70 Torr, respectively. In Fig. 1(a), we also show the total harmonic yields generated by the single-color (SC) 1.6-*μ*m laser alone with the peak intensity of 3.0 × 10^14^ W/cm^2^ under the same macroscopic condition. The harmonics generated with the waveform is about one order higher than that by the SC field.

In Fig. 1(c) and (d), we show the far field harmonics for the two cases above. (Harmonic yields in the two figures have been normalized independently). We can see high harmonics from 70 to 250 eV are well localized close to the propagation axis, their divergence is less than 1 mrad. In comparison with the SC driving laser high harmonics in the same spectral region are quite divergent, making them less useful for applications. One can use an aperture to filter out the divergent harmonics, say those outside 1 mrad. As shown in Fig. 1(b), the result is that harmonic yields from the SC would become two orders smaller than the optimized two-color waveform. Another feature of high harmonics generated by the two-color WF in Fig. 1(a) and (b) is that the spectra are quasi-continuous over a broad spectral range, implying that isolated attosecond pulses may be generated, as to be discussed in Section of “Generation of isolated attosecond pulses in the near and far field”.

We next examine how the harmonic emission varies with the gas pressure. We fixed the waveguide length at 5 mm and considered the gas pressure at 10 and 150 Torr, respectively. The normalized harmonic emissions in the far field are shown in Fig. 1(e) and (f), respectively. For the 10-Torr case, the harmonics are located both near the axis and far off the axis, with the cutoff energy of 250 eV being maintained. For the 150-Torr case, harmonics from 70 to 200 eV are highly concentrated along the axis, but the cutoff energy is greatly reduced. Below we study how the gas pressure and waveguide length affect phase matching for the generation of attosecond bursts, using the method previously^45^ employed for two-color laser pulses.

### Evolution of the driving pulse at different gas pressures

**Figure 2:**
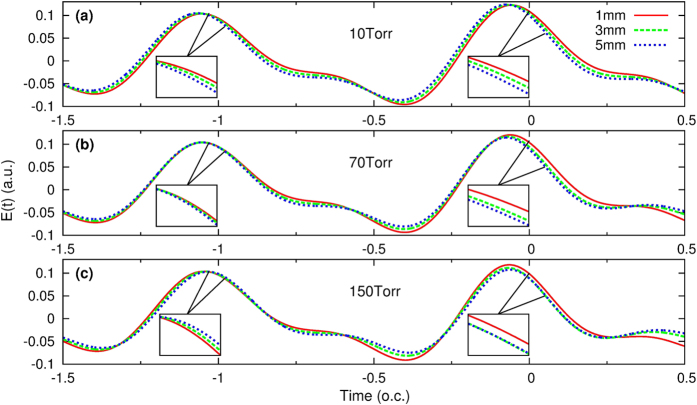
On-axis electric field of the two-color synthesized laser pulse at three gas pressures: (**a**) 10 Torr, (**b**) 70 Torr, and (**c**) 150 Torr. The electric fields are shown at the initial (1 mm), middle (3 mm), and the exit face (5 mm) of the waveguide. Enlarged view of the electric fields is indicated in the inset. Waveguide radius is 125 *μ*m. (o.c. stands for the optical cycle of the 1.6-*μ*m laser).

We show the on-axis electric field in the reference frame at different propagation positions *z* = 1, 3, and 5 mm, for three gas pressures in Fig. 2. The evolution of the electric fields are quite different for each. At 10 Torr, the envelope of the electric field shifts monotonically to the left with increasing *z*. At the low pressure the mode dispersion of the waveguide is dominant and cannot be compensated by the atomic dispersion^45^ alone. This leads to phase mismatch between harmonics generated at different positions of the waveguide to result in diffuse harmonic spectra, as shown in Fig. 1(e). At the optimal pressure of 70 Torr, the electric fields around −1 o.c. (shown in the inset for an enlarged view) are all well overlapped at the three positions, while the electric fields around 0 o.c. (also shown in the inset) are better overlapped between *z* = 3 and 5 mm than between *z* = 1 and 3 mm. Around −1 o.c., the waveguide mode dispersion is compensated only by the neutral atom dispersion. With the increase of time, dispersion due to the plasma is increased, thus around 0 o.c, atomic dispersion is also compensated by the plasma effect^45^. For a single-color laser pulse, this results in a phase mismatch^76^,^77^


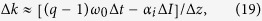


where *q* is the harmonic order, Δ*t* is the shift in time of the peak electric field over a propagation distance Δ*z*, and the second term is dependent of the laser intensity variation Δ*I* and the type of electron trajectory (*α*_*i*_). We use Eq. (19) to estimate the phase mismatch between a two-color driving laser and the generated high harmonics, to decide when the second term can be neglected if only “short”-trajectory emission is considered^77^ and when the laser intensity variation is small, see Table 1. For electrons born around −1 o.c., Δ*t* = −0.01 (−0.006) fs between *z* = 1 and 3 mm (*z* = 3 and 5 mm) in Table 1, the first term in Eq. (19) gives Δ*k* ≈ 1.90 (1.14) *rad*/*mm* for the 250-eV harmonic. The coherence length *L*_*coh*_ = *π*/|Δ*k*| for the 250-eV harmonic between *z* = 1 and 3 mm (*z* = 3 and 5 mm) is thus 1.65 (2.76) mm, which is comparable to or longer than the medium length. For electrons born around 0 o.c., we can similarly calculate the coherence length for the 100-eV harmonic between *z* = 1 and 3 mm (*z* = 3 and 5 mm) as 0.86 (2.07) mm. The former (latter) value is smaller (bigger) than the medium length. The behavior of electric fields at different time moments demonstrates the dynamic nature of phase matching. This explains the buildup of high harmonics due to electrons ionized around −1 o.c. is from *z* = 1 to 5 mm, while high harmonics due to electrons ionized around 0 o.c. only efficiently grow from *z* = 3 to 5 mm (this will be further discussed in the next Section). If gas pressure is increased to 150 Torr, the peak electric fields around −0.5 and 0 o.c. are reduced by the plasma effect, resulting in reduction of cutoff energy, as seen in Fig. 1(f). The overlap of electric fields occurs only around 0 o.c. between *z* = 3 and 5 mm. From such analysis the buildup of harmonics inside the waveguide can be understood even though it is tedious.

### Time-frequency analysis of the evolution of harmonic emission along *z* at the optimal gas pressure

**Figure 3:**
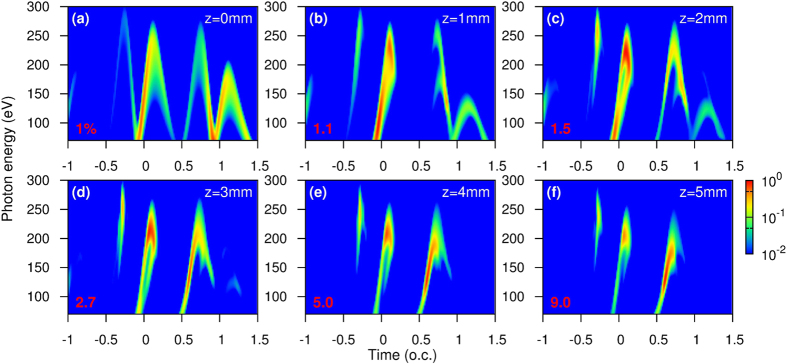
Time-frequency analysis of harmonic emission by the two-color waveform for near-field harmonic integrated over the plane perpendicular to the propagation direction. The normalization factor of harmonic yields in each figure is indicated in red. The results shown are for *z* from 0 to 5 mm in (**a**) to (**f**), respectively. Gas pressure is the optimal value of 70 Torr, radius of the waveguide is 125 *μ*m. (o.c. stands for the optical cycle of the 1.6-*μ*m laser).

In this section we carry out wavelet time-frequency analysis of the evolution of harmonic emission along the waveguide at the optimal gas pressure of 70 Torr. Figure 3 shows the near-field time-frequency harmonic emission, |*A*_*near*_(*t*′, *ω*)|^2^, calculated using Eq. (16), vs the propagation distance *z*. Harmonic yields in each figure were normalized to the maximum value independently. At *z* = 0 mm, the harmonics in Fig. 3(a) reflect features of the single-atom response. From −0.5 to 1.5 o.c., there are four major bursts consisting of “short”- or “long”-trajectory harmonics with positive or negative chirp, respectively. By increasing *z* to 1 mm, see Fig. 3(b), the picture changes dramatically. The two “long”-trajectory bursts from −0.5 to 0.5 o.c. disappear after the propagation. We further look at the two emission bursts: one is between −0.5 and 0 o.c., mostly due to electrons ionized around −1 o.c.; the other is between 0.5 and 1 o.c., caused by electrons ionized around 0 o.c. The first burst has only “short”-trajectory emission at *z* = 1 mm. This burst is maintained in the time domain all the way to *z* = 5 mm, while its intensity grows. This is understood by the good overlap of electric fields around −1 o.c. from *z* = 1 to 5 mm seen in Fig. 2(b), showing good phase matching is achieved. The second burst changes rapidly from *z* = 1 to 3 mm. It only has “long”-trajectory emission at *z* = 1 mm, has both “short”- and “long”-trajectory emissions at *z* = 2 mm, and only has “short”-trajectory emission at *z* = 3 mm. From *z* = 3 to 5 mm, the burst structure is very stable, and its intensity continues to increase. The behaviors for *z* = 1 to 3 mm and for *z* = 3 to 5 mm are totally different but can be explained by the electric fields around −1 o.c. in Fig. 2(b) that good phase matching is only accomplished in the second half of the gas medium. In Fig. 3(f), at the exit face the results show two features: (i) only “short”-trajectory emission is presented, thus the far field harmonics have low divergence in Fig. 1(c), and (ii) three emission bursts are dominant for high harmonics in different photon energy ranges for the generation of isolated attosecond pulses in the near field by proper spectral filtering.

### Generation of isolated attosecond pulses in the near and far field

**Figure 4:**
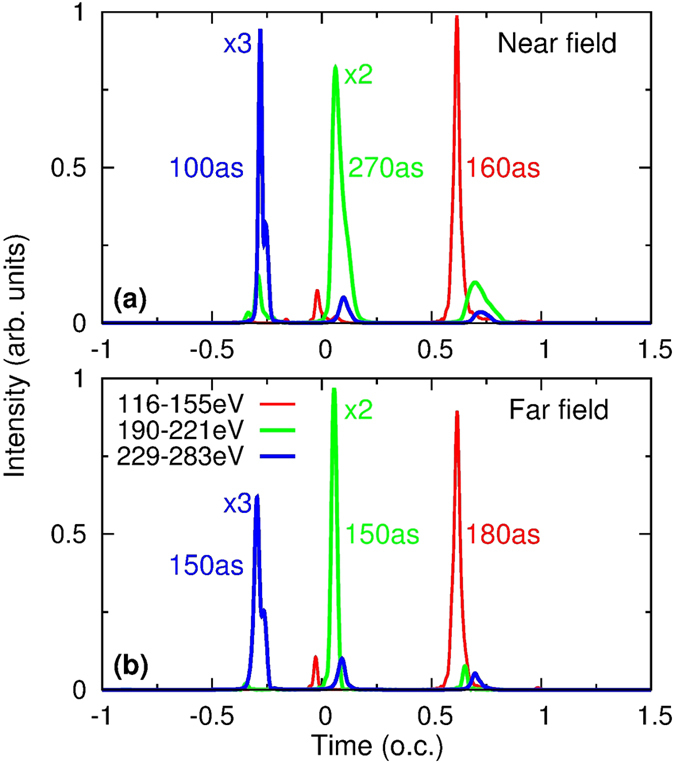
Isolated attosecond pulses (IAPs) at (**a**) near and (**b**) far field after high harmonics have been spectrally filtered in the photon energy ranges indicated. The near-field IAPs are integrated over the whole exit plane. Far-field IAPs are integrated within 2, 1 and 1 mrad from left to right in (**b**), respectively. This indicates that the aperture size varies with the spectral range of high harmonics. The optimal macroscopic conditions are: gas pressure of 70 Torr, waveguide length of 5 mm and radius of 125 *μ*m. Two-color waveform was used. (o.c. means the optical cycle of 1.6-*μ*m laser).

In Fig. 4(a), we show three near-field soft X-ray attosecond pulses that can be synthesized by high harmonics from 116 to 155 eV, 190 to 221 eV, and 229 to 283 eV. They would be able to support isolated attosecond pulses with FWHM durations of 100, 270, and 160 as, respectively. The near-field IAP was calculated by using Eq. (17), and high harmonics were generated by the two-color waveform at the optimal conditions in Fig. 1(a). The three IAPs correspond to three emission bursts in Fig. 3(f). To check the spatial quality of the IAP in the far field and the effect of harmonic propagation in the vacuum, we calculate the far-field IAPs by using Eq. (18) and show the results in Fig. 4(b). The spectral range used to synthesize the IAP is the same as the near-field one. For IAPs centered around −0.3, 0.1, and 0.6 o.c., the angular divergence in the far field was chosen to be 2, 1, and 1 mrad, respectively. We can see from this figure that (i) the intensity of the IAP in the far field is comparable to or even greater than that in the near field, and (ii) the duration of the IAP is close to or even shorter than that in the near field. This example demonstrates that high-intensity and low-divergence soft X-ray IAPs are generated in the far field.

### Generation of high harmonics and isolated attosecond pulses by varying the waveguide radius

In the discussion above, the radius of the waveguide was fixed at 125 *μ*m. Could soft X-ray high harmonics and IAPs be generated if the radius of the waveguide is changed? To answer this question, we chose two radii: 75 and 200 *μ*m (the pulse energy of the incident Gaussian beam is 0.22 and 1.58 mJ, respectively), and searched for the optimal waveguide length and gas pressure to obtain the best cutoff energy and the harmonic yield. For 75 (200) *μ*m, the optimal values of length and pressure are 2 mm and 200 Torr (10 mm and 30 Torr). The incident two-color beam waist is adjusted to ensure that the EH_11_ mode is guided. The input laser pulse energy required for the 200-*μ*m case is much higher than that for the 75-*μ*m one.

**Figure 5:**
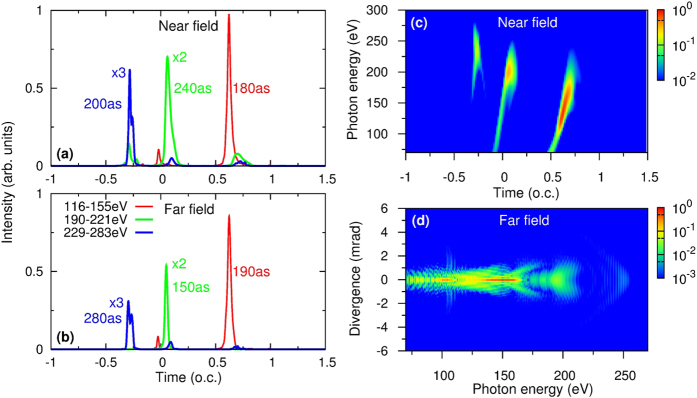
Isolated attosecond pulses (IAPs) at (**a**) near and (**b**) far field. Near-field IAPs are integrated over the exit plane, far-field IAPs are integrated over 2, 1 and 1 mrad from left to right in (**b**). (**c**) Near-field time-frequency analysis of harmonics integrated over the exit plane. (**d**) Divergence of harmonics in the far field. Optimal macroscopic conditions are: gas pressure is 200 Torr, waveguide length is 2 mm and radius is 75 *μ*m. (o.c. is the optical cycle of the 1.6-*μ*m laser).

**Figure 6:**
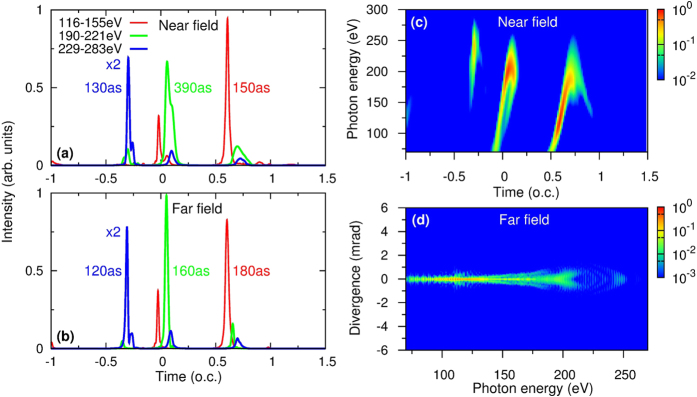
Same as Fig. 5 except that the optimal parameters are: gas pressure is 30 Torr, waveguide length is 10 mm and the radius is 200 *μ*m.

In Fig. 5, we show the time-frequency harmonic emission in the near field, harmonic divergence in the far field, and isolated attosecond pulses in the near and far field for the waveguide radius of 75 *μ*m. Three emission bursts with only “short”-trajectory emission are observed at the exit of the waveguide (*z* = 2 mm) in Fig. 5(c), leading to low divergent harmonics (divergence angle smaller than 1 mrad) in the far field in Fig. 5(d). The low divergent soft X-ray IAPs in the far field [shown in Fig. 5(b)] preserve the intensities and pulse durations of the IAPs in the near field in Fig. 5(a). Similar results are shown in Fig. 6 for the waveguide radius of 200 *μ*m.

## Discussion

In summary, we proposed a method to generate bright soft X-ray isolated attosecond pulses using optimized two-color laser pulses in a hollow waveguide filled with gas. The laser waveform consists of the fundamental laser and its second harmonic has been optimized^42^ for maximal single-atom harmonic yields. In the hollow waveguide its length and radius, as well as the gas pressure, are further optimized to achieve optimal dynamic phase matching. The combined effort allows the optimal generation of intense soft X-ray attosecond pulses. Note that our optimization procedure was performed in a reduced parameter space in which the most important macroscopic parameters have been varied. The scaling of macroscopic parameters may indeed likely^78^, but it needs extensive numerical calculations to verify in the future.

Our simulations demonstrated that high harmonics from XUV to soft X-rays with divergence smaller than 1 mrad can be generated at the optimal macroscopic conditions. In comparison with single-color lasers, the yields were greatly enhanced. The harmonics can be used to produce spatially coherent soft X-ray isolated attosecond pulses with proper spectral filtering. We illustrated the dynamic phase-matching conditions resulting from the interplay among waveguide mode, neutral atomic dispersion, and plasma effect by comparing the time-dependent electric fields at different propagation distances and gas pressures. Time-frequency pictures at optimal conditions showed that the behavior of harmonic emission bursts as a function of propagation distance at different time intervals were quite different, further indicating the dynamic nature of phase matching.

Experimentally, attempts have been made to reach favorite optical waveforms by controlling the multi-color lasers, especially controlling the full phase of a broadband supercontinuum over two octaves^79^. The maturity of waveform-synthesis technology has also been shown by other examples^16^,^36^,^37^,^38^. At the same time, high-repetition-rate lasers of hundreds kHz and MHz lasers^80^,^81^,^82^ are becoming available in the laboratory. By combing with the method suggested in this paper, the increase of harmonic yield or attosecond pulse intensity by several orders is possible. Such all-purpose tabletop isolated attosecond light sources in the soft X-ray region can then be used for time-resolved X-ray spectroscopy in atoms, molecules, and condensed matters.

## Additional Information

**How to cite this article**: Jin, C. *et al*. Optimal generation of spatially coherent soft X-ray isolated attosecond pulses in a gas-filled waveguide using two-color synthesized laser pulses. *Sci. Rep.* **6**, 38165; doi: 10.1038/srep38165 (2016).

**Publisher's note:** Springer Nature remains neutral with regard to jurisdictional claims in published maps and institutional affiliations.

## Figures and Tables

**Table 1:** The instant (*t*_*p*_ around −1 and 0 o.c.) and the strength (*E*_*p*_) of the peak electric field of the laser pulse read from Fig. 2(b) for the optimal pressure of 70 Torr at three propagation positions in the moving frame: *z* = 1, 3 and 5 mm.

Propagation positions	*z* = 1 mm		*z* = 3 mm		*z* = 5 mm	
Time instants	*t*_*p*_ (fs)	*E*_*p*_ (a.u.)	*t*_*p*_ (fs)	*E*_*p*_ (a.u.)	*t*_*p*_ (fs)	*E*_*p*_ (a.u.)
−1 o.c.	−5.602	0.1038	−5.612	0.1041	−5.618	0.1042
0 o.c.	−0.326	0.1206	−0.374	0.1169	−0.394	0.1147

Laser intensity variation is small and can be neglected in the phase mismatch analysis in Eq. (19). (o.c. stands for the optical cycle of the 1.6-*μ*m laser).
